# AAPM medical physics practice guideline 3.b.: Levels of supervision for medical physicists in clinical training

**DOI:** 10.1002/acm2.13258

**Published:** 2021-05-20

**Authors:** Douglas Pfeiffer, Hania Al‐Hallaq, Per Halvorsen, Kristi Hendrickson, Zheng Feng Lu, Melissa Carol Martin, Beth Schueler, Jillian Shuman, Jacqueline E. Zoberi

**Affiliations:** ^1^ Boulder Community Health Boulder CO USA; ^2^ The University of Chicago Chicago IL USA; ^3^ Beth Israel ‐ Lahey Health Burlington MA USA; ^4^ University of Washington Seattle WA USA; ^5^ Therapy Physics, Inc. Signal Hill CA USA; ^6^ Mayo Clinic Rochester MN USA; ^7^ Ascension Via Christi St. Francis Wichita KS USA; ^8^ Washington University School of Medicine St. Louis MO USA

## Abstract

The American Association of Physicists in Medicine (AAPM) is a nonprofit professional society whose primary purposes are to advance the science, education and professional practice of medical physics. The AAPM has more than 8,000 members and is the principal organization of medical physicists in the United States. The AAPM will periodically define new practice guidelines for medical physics practice to help advance the science of medical physics and to improve the quality of service to patients throughout the United States. Existing medical physics practice guidelines will be reviewed for the purpose of revision or renewal, as appropriate, on their fifth anniversary or sooner. Each medical physics practice guideline represents a policy statement by the AAPM, has undergone a thorough consensus process in which it has been subjected to extensive review, and requires the approval of the Professional Council. The medical physics practice guidelines recognize that the safe and effective use of diagnostic and therapeutic radiology requires specific training, skills, and techniques, as described in each document. Reproduction or modification of the published practice guidelines and technical standards by those entities not providing these services is not authorized. The following terms are used in the AAPM practice guidelines: (1) Must and Must Not: Used to indicate that adherence to the recommendation is considered necessary to conform to this practice guideline. (2) Should and Should Not: Used to indicate a prudent practice to which exceptions may occasionally be made in appropriate circumstances.

## 1 INTRODUCTION

The purpose of this practice guideline is to address the levels of supervision necessary for the clinical training of medical physics students, residents, and medical physicists‐in‐training. During the training of these individuals, it is often necessary or desirable for them to perform functions generally performed by Qualified Medical Physicists (QMPs). In such circumstances, the individuals performing these functions **must** be appropriately supervised and the scope of the functions to be performed must be carefully defined. This document does not address the supervision of medical physicist assistants or medical physics extenders, that is, positions not intended to provide a path to becoming a QMP.

It is the responsibility of all individuals to be familiar with federal and state regulations and accreditation requirements regarding supervision that may take precedence over the recommendations in this document.

## 2 DEFINITIONS

### 2.1 Competency

The demonstrated ability to independently perform the medical physics‐related task or function.

### 2.2 Co‐signing or co‐signature

The process of obtaining a second signature or the formal process by which a report produced by a supervised individual is finalized by the supervising QMP. The QMP retains full responsibility for the trainee’s work.

### 2.3 Formal work product

A deliverable or outcome that must be produced as part of the clinical work to complete a project and achieve its objectives.

### 2.4 Medical physics student

An individual enrolled in a masters, doctoral degree‐granting or certificate program from an approved institution (eg, program accredited by one of the organizations recognized by the Council on Higher Education Accreditation, or its successors) in medical physics, physics, or another relevant physical science or engineering discipline.

### 2.5 Medical physicist‐in‐training

An individual who meets the requirements of, and is currently preparing to complete, board certification in one or more of the subfields of medical physics.

### 2.6 Medical physics resident

An individual enrolled in a structured training program designed to educate and train to a level of competency sufficient to practice medical physics independently. This individual **must** have obtained a master’s or doctoral degree in medical physics or a doctoral degree in another relevant physical science or engineering discipline with a certificate in medical physics from an approved institution. An individual enrolled in a Doctorate of Medical Physics (DMP) program also meets this definition of Medical Physics Resident while completing the clinical rotation requirements of the DMP program.

### 2.7 Qualified medical physicist

As defined by AAPM Professional Policy 1.^1^

### 2.8 Supervision

Oversight of and acceptance of responsibility by the QMP for the medical physics‐related work performed by a Trainee or Medical Physics Student:

- General Supervision – The procedure is performed under a QMP’s overall direction and control but the QMP’s presence is not required during the performance of the procedure. Under General Supervision, the training of the personnel who actually perform the procedure and the maintenance of the necessary equipment and supplies are the continuing responsibility of the QMP.
- Direct Supervision – A QMP must exercise General Supervision and be present in the facility and immediately available to furnish assistance and direction throughout the performance of the procedure. It does not mean that the QMP must be present in the room when the procedure is being performed.
- Personal Supervision – A QMP must exercise General Supervision and be present in the room during the performance of the procedure.^2^

### 2.9 Supervisor

A QMP who oversees the medical physics‐related work of a supervised individual in a clinical environment.

### 2.10 Supervised individual

A medical physics student, resident, or medical physicist‐in‐training performing medical physics‐related tasks under the direction of a QMP.

### 2.11 Trainee

The term “trainee” in this document is used to include medical physics residents and medical physicists‐in‐training.

## 3 ROLES OF THE MEDICAL PHYSICS STUDENT, MEDICAL PHYSICS RESIDENT, OR MEDICAL PHYSICIST‐IN‐TRAINING

### 3.1 Medical physics student

Medical physics students **should** be capable of performing basic medical physics tasks with appropriate training, such as the collection of x‐ray generator calibration data for diagnostic / orthovoltage systems or linear accelerator depth dose data. Medical physics students are not expected to analyze or make decisions regarding the data, but may make comments or recommendations to the supervising QMP, and should be involved in the discussion of the data to further their education. If the medical physics student works on clinical medical physics tasks, it **must** be under the personal or direct supervision of a QMP, as deemed appropriate by the QMP. The QMP must provide personal supervision for at least the first performance of a task.

### 3.2 Medical physics resident

Medical physics residents are expected to grow in the degree of responsibility and independence of clinical practice. Early in residency, the resident **should** have responsibilities similar to medical physics students. Personal supervision should be provided for the initial performance of tasks within the medical physics scope of practice.^2^ The resident, with experience and training, **should** progress to analysis of the data and performance of some functions under direct supervision. Late in the residency, the resident **should** be able to function largely as a QMP, with the supervisor balancing the transition from supervision toward independence of the resident during the residency program.

### 3.3 Medical physicist‐in‐training

Medical physicists‐in‐training are expected to grow in the degree of responsibility and independence of clinical practice. With increasing experience, medical physicists‐in‐training **should** be able to function largely as a QMP, with the supervisor balancing the transition from supervision toward independence.

## 4 THE RESPONSIBILITIES OF THE SUPERVISOR

Supervision is a responsibility that **must not** be undertaken lightly. The supervisor **must** assume professional responsibility for the medical physics‐related work done by the supervised individual.

Supervision of a trainee requires regular, high‐quality interactions between the supervisor and the trainee during which medical physics is practiced by the trainee under the guidance of the supervisor. As the trainee grows in professional maturity, it is appropriate for the supervisor to allow the trainee greater responsibility and autonomy, with the understanding that the supervisor **must** still review and co‐sign all formal work products of the trainee. Formal work products could include reports of machine calibrations, shielding designs, treatment plan reviews, patient‐specific quality assurance measurements, treatment record reviews, and equipment evaluations. By co‐signing, the supervisor takes full professional responsibility for the work as if it were their own. A supervised individual who has demonstrated competency through a formal, documented process and has been approved by a supervising QMP may be able to independently complete a task. However, the supervising QMP retains full responsibility for their work and the work product **must** be co‐signed by the QMP. The documented process **must** describe the experience gained through personal, direct, and general supervision of the supervising QMP.

The supervisor **must** have a professional relationship with the supervised individual that allows the supervisor to observe the work of the supervised individual and to correct that work if necessary. The supervisor **should not** commit to the supervisory relationship if:

- The supervised individual and the supervisor work for different employers without a formal agreement outlining the supervisory relationship.
- The supervisor is unable to consistently provide the appropriate level of supervision.
- The supervisor has not committed to assuming full professional responsibility for the supervised individual’s work.
- Except under documented extenuating circumstances (eg, illness or job transition), episodic training sessions, or situations as described in Section 7, a supervisor shall be responsible for no more than 3 full‐time equivalents (FTE) at one time including:
  - No more than two medical physics trainees at one time;
  - No more than two medical physics students at one time;

Supervised individuals who are part‐time in their clinical medical training are counted as their fractional FTE equivalent. For example, if each student is 0.25 clinical FTE and each resident is 0.75 FTE, the maximum of 3 FTE could be maintained with two students and two residents (total 2 FTE in this example).

During a supervisor’s absence, the supervisor is responsible for delegating the required supervision to another QMP. There **must** be a clear description of what tasks may be performed independently by the supervised individual and under what circumstances a delegated QMP **must** cover the supervisory responsibility.

During a supervisor’s absence from work, the supervised individual **must** only be allowed to perform tasks for which that individual has demonstrated and documented formal competency to work independently. The exception is when the supervisor has a coverage arrangement with another QMP and that coverage agreement specifically includes supervision of the supervised individual.

## 5 THE RESPONSIBILITIES OF THE SUPERVISED INDIVIDUAL

The supervised individual **must not** perform clinical medical physics‐related tasks without appropriate supervision or documented competency by the QMP supervisor.

*For supervised individuals in states with professional licensure of medical physicists:* Currently, states that license medical physicists may require an individual working toward becoming a QMP to have a temporary or limited scope license to practice. If a supervised individual holds a temporary license in more than one subfield of medical physics, the supervisor **must** be fully licensed in all of the subfields the supervised individual is training toward (or in which the supervised individual holds a temporary license); otherwise, the supervised individual **must** secure more than one supervisor. However, the individual state mandates for supervision must be followed, as requirements may not be consistent in all states. It is the responsibility of the supervised individual to ensure that his or her supervisor(s) are licensed in the subfield at the time of the application for a temporary license.

## 6 A GENERAL PROGRESSION OF SUPERVISION FOR TRAINEES

There **must** be a progression from personal to general supervision for trainees. A supervision plan **must** be available and acknowledged by the trainee and the supervisor. Competency performance reviews **should** be completed and documented for each activity.

### 6.1 Supervision plan

A supervision plan **must** be formally adopted and document well‐defined progression of levels of responsibility for trainees. The assignment of trainee to supervisor (ie, which supervisor is responsible for which trainee) must be clear to all involved with clear lines of communication established.

As an example, a plan consisting of the following would meet the intent of this document:

- Personal supervision for the first 6 months,
- Direct supervision for the remainder of the first year, and
- General supervision in the second year.

Note that the supervisor is not relieved of the responsibility to review and co‐sign reports and retains full responsibility for their content.

### 6.2 Considerations in developing a supervision plan

Prior to determining the assignment of work, the facility **must** have a process to facilitate documentation of a co‐signature and to define the conditions under which a supervised individual can practice.

It is essential that the QMP determine whether a supervised individual may visit a facility alone for a specific activity. The QMP **must** determine whether the QMP should also personally participate in the initial visit (or more as required) to a facility to address unanticipated issues. A supervised individual **must not** be sent alone to perform a procedure that is not in the scope of practice for the role of the supervised individual, or for which the supervised individual has not demonstrated formal competency.

It is also essential that the plan delineates how a supervised individual may proceed from personal supervision to direct supervision and from direct supervision to general supervision. This may be task dependent with higher‐level functions or critical patient care functions requiring more training and experience to be performed under personal or direct supervision than low‐level functions. Examples of tasks requiring higher levels of supervision are measurements performed on clinical teletherapy or imaging equipment to determine the output or beam characteristics. Subsequent verification of radiation outputs could be considered a lower‐level function. The progress plan with milestones to be achieved by the trainee **should** be documented.

## 7 MEDICAL PHYSICS RESIDENCY PROGRAMS

The Residency Program Director must be a QMP and should have at least 5 years of full‐time experience beyond residency.^3^, ^4^ All faculty members of a medical physics residency program **should** be fully qualified (including being licensed if practicing in a state with a medical physics licensure law) in the clinical activities that they supervise. The Residency Program Director who is on the faculty of a medical physics residency program may delegate day‐to‐day supervision of residents:

- To colleagues on the faculty who are QMPs or
- To other qualified faculty or staff members only if:
  ▪ A QMP in the appropriate subfield of medical physics co‐signs each item of work produced by the resident, and
  ▪ The formal supervisor shares with the delegate the authority to observe and correct the work of the resident.

The Residency Program Director exercises general supervision over all residents in the program. The Residency Program Director delegates responsibility for supervision to faculty members of the residency program by assignment of residents for a task or time period. Faculty members who have responsibility for supervision of a resident may further delegate authority for supervision to other faculty members who are QMPs for the tasks to be performed by the resident.

It is reasonable for one faculty member to be the formal supervisor of all residents, even more than two at a time, and to delegate day‐to‐day supervision to other qualified faculty members. Supervision cannot be delegated from a QMP to a resident; however, it is entirely appropriate for a more experienced resident to assist in the training of another resident. A QMP must supervise the training of one resident by another, must provide personal supervision of each resident during the first performance of a task at minimum, and must co‐sign the joint work product of the two residents, who must also sign as residents. The supervising QMP is still ultimately responsible for the work of both residents.

## 8 RECOMMENDATIONS

Clinical practice environments must balance the need for competent staff with the need to train new professionals and provide services in a safe yet cost‐effective manner. The guidelines presented in this document provide a framework for the appropriate supervision and scope of responsibilities for medical physics students, residents, and medical physicists‐in‐training.

Specifically:

- The supervisor **must** assume professional responsibility for the medical physics‐related work done by the supervised individual.
- A supervision plan **must** be formally adopted and document well‐defined progression of levels of responsibility for trainees.
- Except under certain circumstances, as outlined in Section 4.d., a supervisor **must not** supervise:
  - More than two medical physics trainees at one time;
  - More than two medical physics students at one time; and
  - More than three full‐time equivalents per supervisor.

## TG‐243 Members/Authors

Seibert, PhD, FAAPM, FACR, FSIIM, Chair

Jessica B. Clements, MS, FAAPM

Per H. Halvorsen, MS, FAAPM, FACR

Michael G. Herman, PhD, FAAPM, FACMP

Melissa C. Martin, MS, FAAPM, FACR, FACMP

Jatinder Palta, PhD, FAAPM, FACR, FASTRO, FACMP

Douglas E. Pfeiffer, MS, FAAPM, FACR

Robert J. Pizzutiello, Jr. MS, FAAPM, FACR, FACMP

Beth A. Schueler, PhD, FAAPM, FACR

S. Jeff Shepard, MS, FAAPM

Lynne A. Fairobent, AAPM Staff

**AAPM Subcommittee on Practice Guidelines** — AAPM Committee responsible for sponsoring the draft through the process.

Dan Pavord, MS, FAAPM, Chair

Muthana S.A. L. Al‐Ghazi, PhD, FAAPM, FCCPM, FIOMP

Nathan C. Busse, MS

Leigh A. Conroy, PhD

Ryan F. Fisher, PhD

Jonas D. Fontenot, PhD, FAAPM

Mark W. Geurts, MS

Eric Gingold, PhD, FAAPM

Per H. Halvorsen, MS, FAAPM, FACR

Mary A. Keenan, DMP, Vice‐Chair Imaging

Steven P. McCullough, PhD

Arthur J. Olch, PhD, FAAPM, Vice‐Chair Therapy

Douglas E. Pfeiffer, MS, FAAPM, FACR

Susan L. Richardson, PhD, FAAPM

John M. Wait, MS

Ping Xia, PhD, FAAPM

Nicholai E. Wingreen, AAPM Staff

## AUTHOR CONTRIBUTIONS

This guideline was reviewed and updated by the Medical Physics Practice Guideline Task Group 340 of the Professional Council of the AAPM. Each author reviewed recent literature on the topic and offered opinions on and language for updates to be made to MPPG 3.a. They also reviewed and applied comments from the full AAPM membership to the document.

## CONFLICT OF INTEREST

The Chair of Task Group No. 340 ‐ MPPG 3.b: Levels of supervision for medical physicists in clinical training (TG340) has reviewed the required Conflict of Interest statement on file for each member of TG‐340 (3 instances) and determined that disclosure of potential Conflicts of Interest is an adequate management plan. Disclosures of potential Conflicts of Interest for each member of TG340 are found at the close of this document.

## TG‐340 Members

Douglas Pfeiffer MS, FAAPM, FACR, Chair

Boulder Community Health

Hania Al‐Hallaq PhD, FAAPM

The University of Chicago

Per Halvorsen MS, FAAPM, FACR

Beth Israel ‐ Lahey Health

Kristi Hendrickson PhD, FAAPM

University of Washington

Zheng Feng Lu PhD, FAAPM

University of Chicago

Melissa Carol Martin MS, FAAPM, FACR, FACMP

Therapy Physics, Inc.

Beth Schueler, PhD, FAAPM, FACR

Mayo Clinic

Jillian Shuman DMP

Ascension Via Christi St. Francis

Jacqueline E. Zoberi PhD, FAAPM

Washington University School of Medicine
